# Therapeutic Effects of an Anti-sialyl Lewis X Antibody in a Murine Model of Allergic Asthma

**DOI:** 10.3390/ijms22189961

**Published:** 2021-09-15

**Authors:** Wei Xiong, Wenxin Liu, Shogo Nishida, Daichi Komiyama, Wei Liu, Jotaro Hirakawa, Hiroto Kawashima

**Affiliations:** Laboratory of Microbiology and Immunology, Graduate School of Pharmaceutical Sciences, Chiba University, Chiba 260-8675, Japan; xiongwei@chiba-u.jp (W.X.); lwxalish@yahoo.com (W.L.); la.vita.e.bella.for13@gmail.com (S.N.); cata4920@chiba-u.jp (D.K.); ryuyi518@yahoo.co.jp (W.L.); j.hirakawa@icloud.com (J.H.)

**Keywords:** druggable targets, sialyl Lewis X, antibody, asthma, leukocyte infiltration

## Abstract

Asthma is an allergic disease that causes severe infiltration of leukocytes into the lungs. Leukocyte infiltration is mediated by the binding of sialyl Lewis X (sLe^x^) glycans present on the leukocytes to E-and P-selectins present on the endothelial cells at the sites of inflammation. Here, we found that mouse eosinophils express sLe^x^ glycans, and their infiltration into the lungs and proliferation in the bone marrow were significantly suppressed by an anti-sLe^x^ monoclonal antibody (mAb) F2 in a murine model of ovalbumin-induced asthma. The percentage of eosinophils in the bronchoalveolar lavage fluid and bone marrow and serum IgE levels decreased significantly in the F2-administered mice. Levels of T helper type 2 (Th2) cytokines and chemokines, involved in IgE class switching and eosinophil proliferation and recruitment, were also decreased in the F2-administered mice. An ex vivo cell rolling assay revealed that sLe^x^ glycans mediate the rolling of mouse eosinophils on P-selectin-expressing cells. These results indicate that the mAb F2 exerts therapeutic effects in a murine model of allergen-induced asthma, suggesting that sLe^x^ carbohydrate antigen could serve as a novel therapeutic target for allergic asthma.

## 1. Introduction

Bronchial asthma is the most common chronic airway disease with over 250 million patients worldwide [1]. It is characterized by chronic airway inflammation and remodeling, which result in a variety of respiratory symptoms such as wheezing, heavy cough, and shortness of breath [2]. The severity of asthma varies from patient to patient depending on genetic as well as environmental factors [2]. In most cases, asthma originates from an exposure to an environmental allergen followed by IgE-dependent sensitization [3]. Although asthma can be divided into several different subphenotypes, T helper type 2 (Th2) response-driven allergic asthma is the most prominent [4].

Asthma can only be controlled rather than cured. However, since the pathogenesis of asthma, especially allergic asthma, has been well researched during the last few decades, an increasing number of advanced treatments are being developed. According to the international guidelines of the Global Initiative for Asthma, the basic treatment of asthma involves administration of inhaled corticosteroids (ICS) [5]. If the symptoms remain uncontrolled after the ICS treatment, administration of a long-acting β_2_-agonist needs to be added to the treatment [6]. If such treatments cannot completely control the asthmatic symptoms, additional treatments are induced to control the severe asthma. One such additional method includes the administration of macrolide antibiotics that have immunomodulatory and anti-inflammatory effects in addition to their antimicrobial activity [7]. Recently, monoclonal antibody (mAb) drugs have been widely used in the treatment of various diseases, because they can aim for specific targets in a defined pathway. Several types of mAbs have been developed for the treatment of asthma. Anti-IgE mAb (omalizumab) was the first successful mAb that helps patients with asthma through prevention of mast cell and basophil degranulation by blocking IgE binding to FcεRI [8,9]. However, because of the complex pathology of asthma, they cannot be effective in every patient, which makes it necessary to develop precise treatments for specific phenotypes of asthma. For example, anti-IL-5 mAb (Reslizumab) and anti-IL-5Rα mAb (Benralizumab) have been developed to treat eosinophilic asthma because IL-5 is an important cytokine for the maturation and activation of eosinophils [10]. Although anti-IL-5 or anti-IL-5Rα mAbs have therapeutic effects on patients with allergic asthma and eosinophilia, no therapeutic effects were observed in patients with moderate or neutrophilic asthma [11]. 

In most tissues, leukocytes are generally recruited through a cascade of four steps: rolling, adhesion, crawling, and transmigration [12]. In allergic asthma, inflammatory mediators are released after allergen exposure, which induce the expression of E- and P-selectins on the endothelial surface at the site of inflammation. These two selectins have similar functions in mediating leukocyte infiltration. The sialyl Lewis X (sLe^x^) glycans on P-selectin glycoprotein ligand 1 (PSGL-1), which is expressed on the surface of leukocytes, bind to P- and E-selectins and capture the leukocytes in the blood flow, allowing them to roll along the direction of the blood flow [13]. It has been proven that without the binding of PSGL-1 to E- or P-selectin, leukocytes cannot start rolling on the endothelial surface and the subsequent cascade of events cannot happen [14,15], leading to suppressed leukocyte infiltration to the sites of inflammation [16]. Therefore, the leukocyte rolling step is a reasonable target for the treatment of asthma.

Based on the abovementioned mechanisms for leukocyte infiltration, we hypothesized that targeting sLe^x^, the ligand for P- and E-selectin, with specific anti-glycan antibodies would be a potential therapy for asthma. In our previous study, we developed an anti-sLe^x^ mAb, termed F2, that specifically reacts with sLe^x^ glycans expressed in both humans and mice [17]. The glycan array analysis showed that the mAb F2 specifically bond to sLe^x^, but not to other glycans. Administration of F2 into mice significantly blocked lymphocyte homing to peripheral lymph nodes and suppressed leukocyte infiltration in a murine contact hypersensitivity model. Based on these findings, we hypothesized that mAb F2 should have therapeutic effects against asthma. In this study, to explore whether sLe^x^ could be a target for the treatment of allergic asthma, we investigated the expression of sLe^x^ in mouse eosinophils using the mAb F2 and examined its therapeutic effects in a murine model of allergen-induced asthma.

## 2. Results

### 2.1. SLe^x^ Deficiency in Eosinophils Resulted in Diminished Infiltration into the Lungs

**Figure 1:**
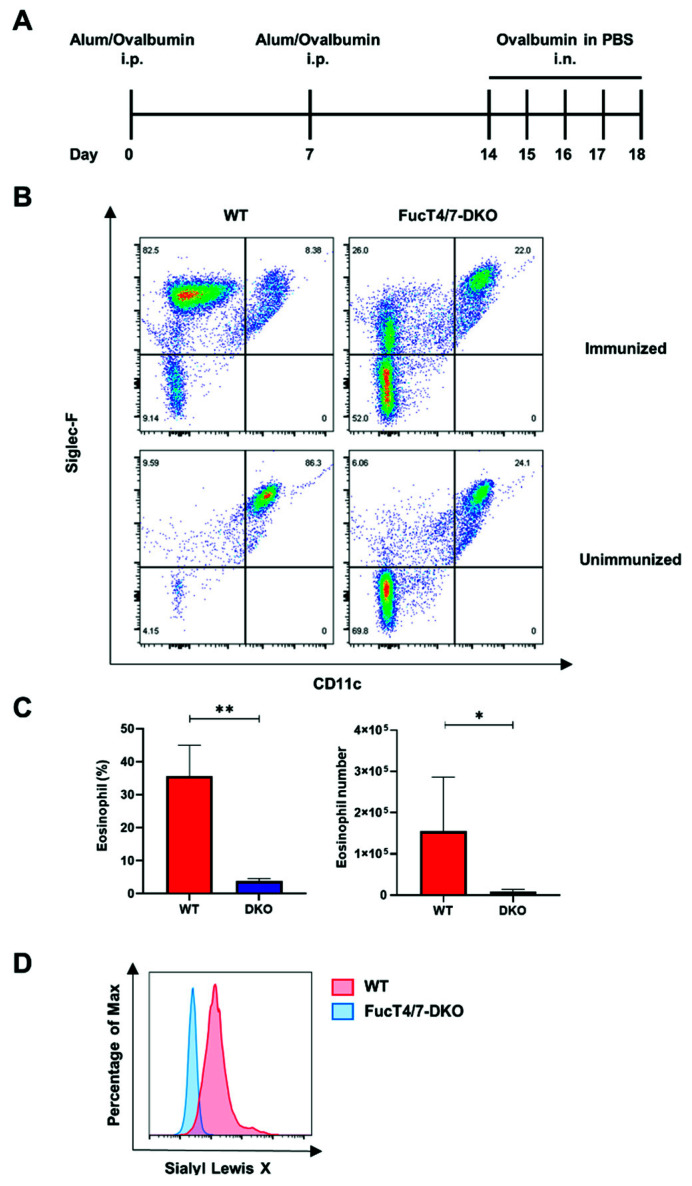
SLex deficiency of eosinophils resulted in diminished infiltration into the lungs. (**A**) The schedule of the immunization. Intraperitoneal injection of 100 μg OVA emulsified with ImjectAlum was used for sensitization on days 0 and 7. The challenge was performed under isoflurane anesthesia by intranasal injection of 100 μg OVA once a day for 5 days starting days 14. The mice were sacrificed 24 h after the final challenge. (**B**) The harvested BALF was analyzed using flow cytometry. The eosinophils were defined as CD45^+^Siglec-F^+^CD11c^−^. The alveolar macrophages were defined as CD45^+^Siglec-F^+^CD11c^+^. The number in the corner shows the percentage of each cell population. (**C**) The percentage of eosinophils in the whole leukocytes and the number of eosinophils were calculated. At least four mice were included in each group. The experiments were conducted twice independently. (**D**) The expression of sLe^x^ on the eosinophils was determined using mAb F2. * *p* < 0.05, ** *p* < 0.01 A *p*-value of less than 0.05 was considered significant.

To investigate the role of sLe^x^ in eosinophil infiltration, we induced allergic asthma in WT and FucT-IV/VII DKO [18] mice lacking sLe^x^ according to the schedule shown in Figure 1A. Flow cytometry was performed using BALF collected from ovalbumin (OVA)-administered mice 24 h after the last challenge. As shown in Figure 1B, OVA-administered WT mice had severe eosinophil infiltration in the BALF compared to that in untreated mice. The CD11c^–^/Siglec-F^+^ compartment represents eosinophils. In contrast, eosinophil infiltration was strongly diminished in OVA-administered DKO mice compared to that in WT mice. The percentage of eosinophils among CD45^+^ leukocytes (Figure 1C, left), and the number of infiltrated eosinophils in the BALF (Figure 1C, right) were both significantly decreased.

The expression of sLe^x^ on eosinophils was examined using mAb F2, which reacts well with sLe^x^ expressed in both humans and mice [17]. As expected, the eosinophils from WT mice showed high expression of sLe^x^, while eosinophils from the DKO mice lacked its expression (Figure 1D). Since the binding of sLe^x^ to selectins is important for the infiltration of other leukocytes, these results suggest that a similar mechanism might play a role in eosinophil infiltration to the sites of inflammation.

### 2.2. Administration of mAb F2 Suppressed Eosinophil Infiltration

**Figure 2:**
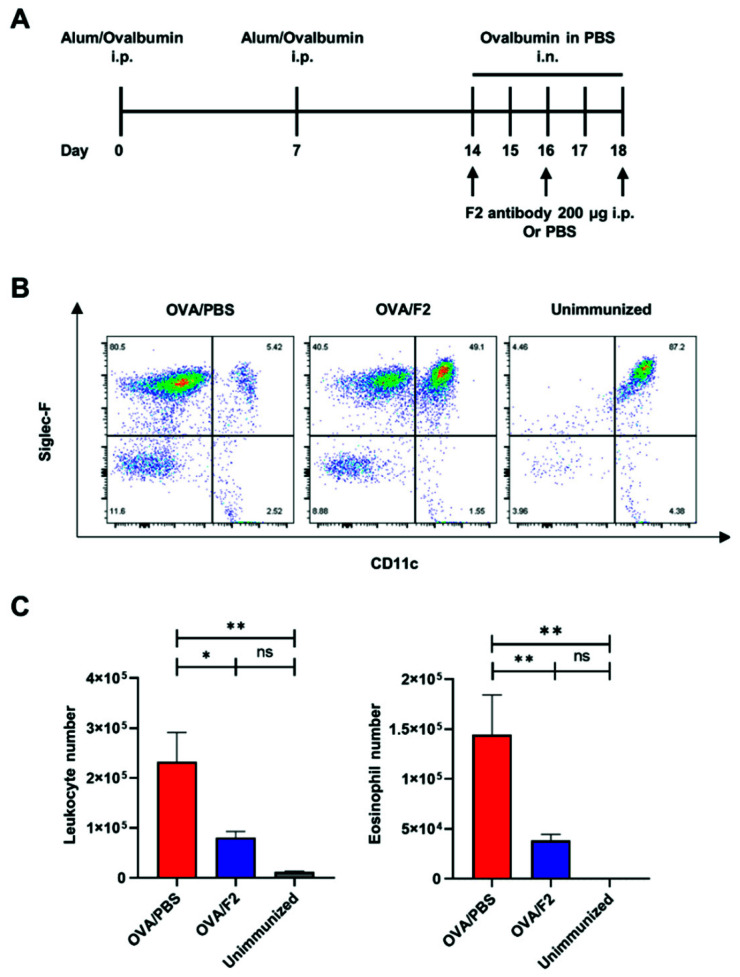
Administration of mAb F2 suppressed eosinophil infiltration. (**A**) The schedule of the immunization. OVA was used as the allergen emulsified with ImjectAlum for sensitization. The challenge was performed under isoflurane anesthesia by intranasal injection. Two hundred μg mAb F2 or 200 μL PBS was injected intraperitoneally on days 14, 16, and 18. The first injection was performed 2 h before the first intranasal challenge. (**B**) The harvested BALF was analyzed using flow cytometry. The eosinophils were defined as CD45^+^Siglec-F^+^CD11c^−^. The alveolar macrophages were defined as CD45^+^Siglec-F^+^CD11c^+^. The number in the corner shows the percentage of each cell population. (**C**) The total number of leukocytes and eosinophils from the BALF obtained from the control, F2-administered, and unimmunized groups of mice. The data were pooled from five independent experiments. The control (*n* = 25), F2-administered (*n* = 28), and unimmunized groups (*n* = 15) are shown in the figure. * *p* < 0.05, ** *p* < 0.01. A *p*-value of less than 0.05 was considered significant.

To further define the role of sLe^x^ in eosinophil infiltration, we examined the effects of mAb F2 administration in the asthma model (Figure 2A). As shown in Figure 2B, mice from the control group showed severe eosinophil infiltration into the BALF compared to that in unimmunized mice. In contrast, the mice from the F2-administered group had suppressed eosinophil infiltration compared to that in the mice from the control group. The number of CD45^+^ leukocytes in the BALF (Figure 2C, left) and the number of eosinophils within leukocytes (Figure 2C, right) both decreased significantly in the F2-administered group, which suggests that eosinophil infiltration relied on sLe^x^.

### 2.3. Decreased Eosinophil Infiltration and Relieved Allergic Responses in mAb F2-Administered Mice

**Figure 3:**
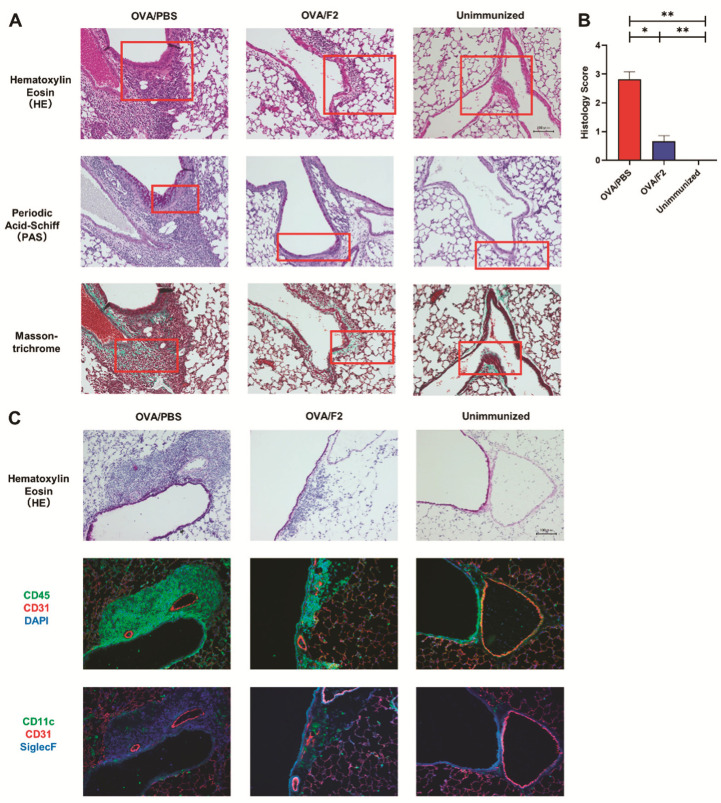
Decreased eosinophil infiltration and relieved allergic symptoms in mAb F2-administered mice. (**A**) Representative images are shown. Three different staining protocols were used to examine the leukocyte infiltration (H&E, upper), airway mucin production (PAS, middle), and airway fibrosis (MT, bottom). In the H&E staining, the cell nuclei are stained with blue, while the airway tract is stained with pink. In the PAS staining, the cell nuclei are stained with blue, while the polysaccharides of the mucin are stained with purple. In the MT staining, the collagen in the airway fibrosis is stained with green. Representative areas are marked with red. Scale bar, 100 μm. (**B**) The histology score of lungs were determined by the severity of leukocyte infiltration. Each group contained 3–4 mice. At least three areas in each section were evaluated. (**C**) Immunofluorescence was performed using different combinations of fluorescence-conjugated antibodies. After induction of asthma, a significant number of CD45^+^ leukocytes infiltrated around CD31^+^ blood vessels and most of the leukocytes infiltrated were Siglec-F^+^ eosinophils (OVA/PBS). The leukocyte infiltration was significantly suppressed by F2 administration (OVA/F2). Scale bar, 100 μm. * *p* < 0.05, ** *p* < 0.01. A *p*-value of less than 0.05 was considered significant.

To further determine the infiltration of eosinophils in the lung tissues, the lungs were collected from OVA-administered mice treated with or without mAb F2 and examined using tissue staining. Paraffin sections of the lungs were subjected to H&E (Figure 3A, top), periodic acid-Schiff (PAS) (Figure 3A, middle), and Masson’s trichrome staining (MT) (Figure 3A, bottom). From the results of H&E staining, severe infiltration of leukocytes was observed in the control group, while it was significantly suppressed in the F2-administered group. The airway mucin was stained violet in PAS staining, and the airway collagen was stained green in Masson-trichrome staining. Overproduction of mucin and airway fibrosis were both suppressed significantly in the F2-administered group compared with those in the control group. The histology scores of lungs from the F2-administered group decreased compared with those from the PBS group (Figure 3B).

To define the cell type of the infiltrated leukocytes, immunofluorescence was performed using frozen sections. H&E staining was also performed to detect leukocyte infiltration (Figure 3C, top). The leukocytes were stained with anti-mouse-CD45 mAb, while the endothelial cells of the blood vessels were stained with anti-mouse-CD31 mAb (Figure 3C, middle). The alveolar macrophages were stained with anti-mouse-CD11c mAb, and the eosinophils were stained with anti-mouse-Siglec-F mAb (Figure 3C, bottom). The results in Figure 3B show that most of the infiltrated leukocytes were eosinophils, which is consistent with the results of flow cytometry. Interestingly, the fluorescence density decreased from the blood vessel to the airway, suggesting that eosinophils infiltrated from the blood vessels to the airway and the blockage of sLe^x^ with mAb F2 inhibited eosinophil infiltration.

### 2.4. Administration of mAb F2 Suppressed Allergic Immune Response in the Lungs

**Figure 4:**
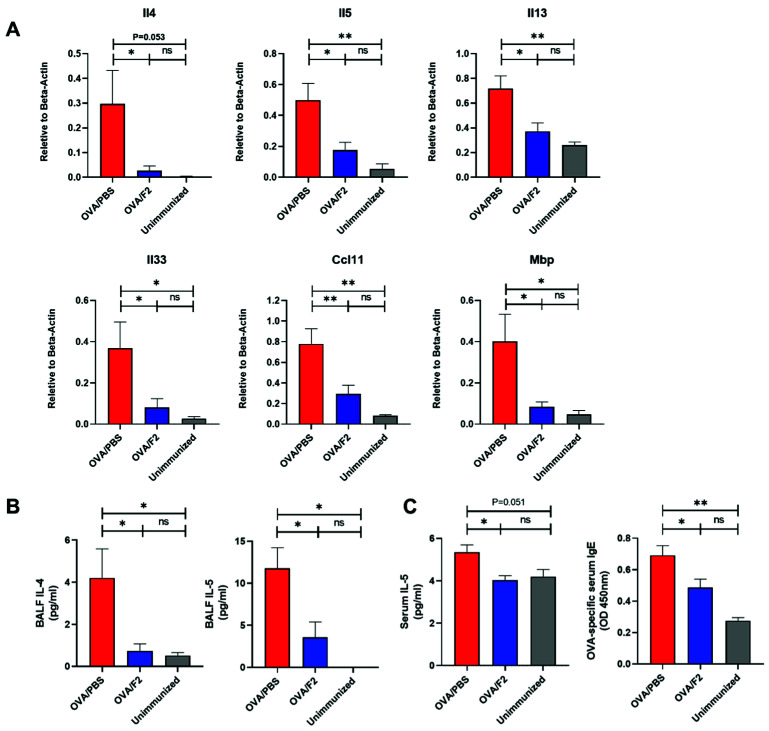
Administration of mAb F2 suppressed the allergic immune response in the lungs and serum. (**A**) The mRNA expression in the lung of IL-4, IL-5, IL-13, IL-33, CCL11, and MBP was examined using quantitative-PCR. Samples from 2 independent experiments were examined. (**B**) The concentration of the Th2 cytokine IL-4 and IL-5 in the BALF was determined by ELISA. (**C**) The concentration of IL-5 and the amounts of OVA-specific antibodies in the serum were determined using ELISA. The experiment was repeated twice independently. * *p* < 0.05, ** *p* < 0.01. A *p*-value of less than 0.05 was considered significant.

The blockage of sLe^x^ resulted in not only a decrease in eosinophils, but also the attenuation of allergic responses, including mucin overproduction and lung fibrosis, which led us to further investigate the changes in the expression of Th2 cytokines and chemokines. We extracted total RNA from the lung tissue of mice from both the control and the F2-administered groups in the allergic asthma model and examined the changes in the Th2 cytokines IL-4, IL-5, and IL-13, eosinophil-recruiting chemokine CCL11 [19] and allergic asthma-related cytokine IL-33 [20] using RT-qPCR (Figure 4A). The expression of all these cytokines and chemokines was observed to be lower in the F2-administered group than in the control group. In addition, the expression of major basic protein (MBP), the main granule protein from eosinophils [21], was decreased in the F2-administered mice. These data suggest that blockade of eosinophil infiltration by mAb F2 leads to the suppression of the allergic response cascade in the asthma model. 

To further determine the change in Th2 response at the protein level, ELISA was performed. As shown in Figure 4B, the concentration of IL-4 and IL-5 in the BALF decreased in the F2-administrated group. Furthermore, the concentration of IL-5 and the amount of OVA-specific IgE in the serum also decreased in the F2-administered group (Figure 4C). These data collectively provide evidence that the allergic responses were suppressed by F2 administration.

### 2.5. Blockade of sLe^x^ Resulted in Reduced Differentiation of Eosinophils in the Bone Marrow

**Figure 5:**
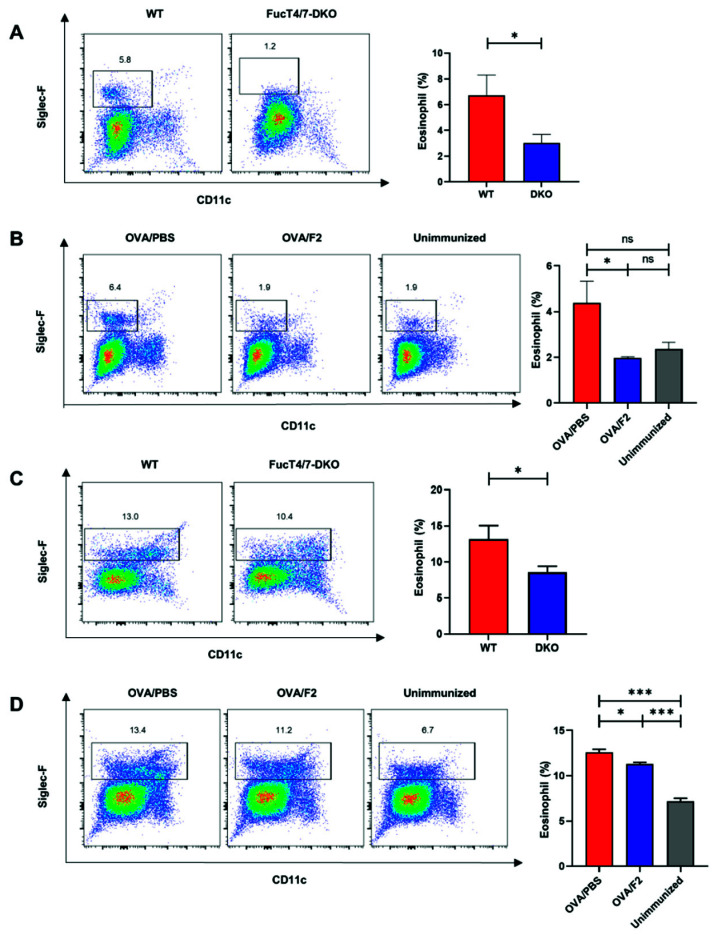
Attenuated allergic responses resulted in reduced differentiation of eosinophils in the bone marrow. (**A**,**B**): The percentage of eosinophils in the blood was determined using flow cytometry. The CD45^+^Siglec-F^+^CD11c^−^ cells were defined as eosinophils. The percentage of eosinophils in the blood in the OVA-administered WT and DKO mice (**A**) or that in the OVA-administered control (OVA/PBS), OVA-administered F2 (OVA/F2), and unimmunized groups of mice (**B**–**D**): The percentage of eosinophils in the bone marrow was determined using flow cytometry. The CD45^+^Siglec-F^+^CD11c^−^ and CD45^+^Siglec-F^+^CD11c^+^ bone marrow cells were defined as eosinophils [22]. The percentage of eosinophils in the bone marrow in the OVA-administered WT and DKO mice (**C**) or that in the OVA-administered control (OVA/PBS), OVA-administered F2 (OVA/F2), and unimmunized groups of mice (**D**). At least four mice were included in each group. The experiments were conducted twice independently. * *p* < 0.05, *** *p* < 0.001. A *p*-value of less than 0.05 was considered significant.

The suppressed eosinophil infiltration to the lung tissue has a possible risk of inducing eosinophilia in the blood. To examine this possibility, the peripheral blood from WT and DKO mice was analyzed using flow cytometry (Figure 5A). Surprisingly, however, the percentage of eosinophils in the blood from the immunized DKO mice was significantly lower than that from the WT mice, indicating that eosinophilia was not observed in the DKO mice. Furthermore, the percentage of eosinophils was significantly increased in the peripheral blood of the immunized control group, but not in the F2-administered group compared to that observed in unimmunized mice (Figure 5B). These results suggest that eosinophil differentiation was upregulated in the bone marrow after the onset of OVA-induced allergic asthma, but was downregulated by the deficiency or blockage of sLe^x^.

To examine this possibility, we examined whether OVA-induced allergic asthma influenced the number of eosinophils in the bone marrow. As shown in Figure 5C, the percentage of eosinophils in the bone marrow of the DKO mice was significantly lower than that of the WT mice (Figure 5C). The percentage of eosinophils in the bone marrow of the F2-administered mice was also suppressed compared to that in the control mice (Figure 5D). Compared with the unimmunized mice, the mice with OVA-induced allergic asthma (OVA/PBS in Figure 5D) showed a higher percentage of eosinophils in the bone marrow, supporting the idea that eosinophil differentiation was upregulated in the bone marrow in asthma. These results suggest that the deficiency or blockade of sLe^x^ on eosinophils suppressed eosinophil differentiation in the bone marrow of mice with asthma and led to a decrease in the number of eosinophils in the blood, which might be related to the attenuation of allergic responses.

### 2.6. SLe^x^ Played a Vital Role in the Rolling of Eosinophils by Binding to p-Selectin

**Figure 6:**
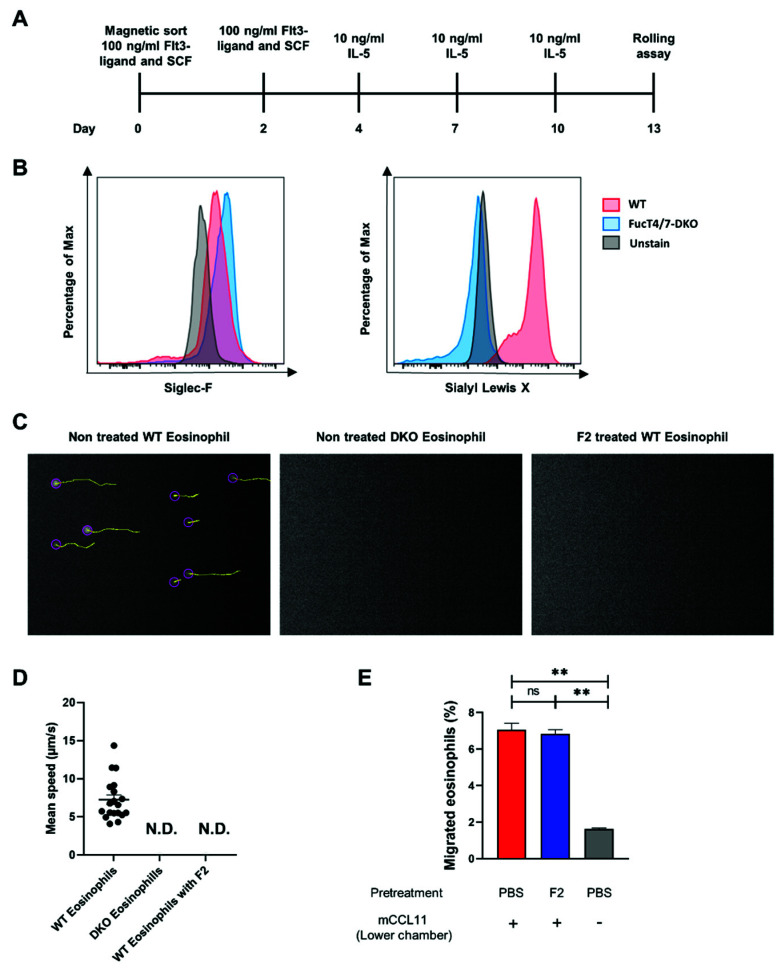
SLex mediated rolling of eosinophils on P-selectin-expressing cells without affecting the migration. (**A**) The schedule of eosinophil induction. The mouse hematopoietic progenitor cells were isolated from the bone marrow using the Mojosort^TM^ hematopoietic progenitor cell isolation kit. FLT3-Ligand and SCF were added to the culture medium on days 0 and 2. The medium was partly exchanged with medium supplemented with IL-5 on days 4, 7, and 10. (**B**) The harvested eosinophils were analyzed using flow cytometry. The mature eosinophils were defined as CD45^+^Siglec-F^+^CD11b^+^CCR3^High^. The experiment was independently repeated twice. (**C**) The rolling assays were performed, and the videos were taken using a CMOS camera equipped on a microscope at the speed of 20 photos per second for 30 s. In total, the stuck of 600 pictures were analyzed using Trackmate in Fiji ImageJ after subtracting the background. The rolling cells are circled with purple, and their rolling tracks are drawn in yellow by the software. The experiments were repeated twice independently. (**D**) The speed of the rolling cells was calculated using the Trackmate in Fiji ImageJ. In the WT eosinophil group, most of the cells moved between 5 and 15 μm/s. No rolling cells were detected in the DKO eosinophil (DKO Eosinophils) and F2-treated WT eosinophil groups (WT Eosinophils with F2). (**E**) A migration assay was performed using transwells, and the percentage of migrated eosinophils into the lower chamber was shown. Cells from the murine bone marrow was pretreated with or without F2 mAb and used for the migration assay. The lower chamber contained mCCL11 or medium only. ** *p* < 0.01. A *p*-value of less than 0.05 was considered significant.

The expression of sLe^x^ on eosinophils has been determined, but whether eosinophil infiltration into the site of inflammation shares a similar mechanism with other leukocyte infiltration needs further confirmation. The rolling assay, which forces leukocytes to roll on a cell layer, is a suitable tool to examine the initial step of leukocyte infiltration mediated by sLe^x^ glycans and selectins. To perform a rolling assay using eosinophils, we induced the eosinophils ex vivo [23] to harvest large numbers of eosinophils. Briefly, hematopoietic stem cells were enriched with a magnetic column and cultured in the presence of FLT3-ligand and SCF, followed by the addition of IL-5 to induce differentiation into eosinophils (Figure 6A). The induction efficiency of eosinophils was examined immediately before the rolling assay using flow cytometry (Figure 6B). The cells from both the WT and DKO mice expressed the eosinophil marker Siglec-F. Bone marrow-derived eosinophils from the WT mice expressed high levels of sLe^x^, while those from DKO mice failed to express sLe^x^.

The rolling assay was performed using eosinophils from WT mice (Figure 6C, left), those from DKO mice (Figure 6C, middle), and those from WT mice that had been treated with mAb F2 (Figure 6C, right). CHO cells expressing P-selectin were used as the cell layer to mimic the endothelial cells expressing P-selectin at the sites of inflammation. As shown in Figure 6C, rolling cells are circled in purple, and the tracks of rolling are shown as yellow lines. Eosinophils from WT mice rolled at a stable speed between 5 and 10 μm/s on the cell layer, whereas eosinophils from DKO mice and those from WT mice that had been treated with F2 failed to roll on the cell layer (Figure 6D, also shown in Supplementary Videos S1–S3). 

To determine the possibility that F2 might also affect the migration of eosinophils, a migration assay was set up using an uncoated transwell system. Murine CCL11 induced notable migration of eosinophils through the transwell into the lower chamber, while the treatment with F2 did not affect the migration of eosinophils (Figure 6E). Collectively, these results indicate that sLe^x^ is critical for the rolling of eosinophils on P-selectin expressing cells, but not chemokine-induced transmigration.

## 3. Discussion

In our study, asthma was first induced in WT mice and FucT-IV/VII DKO mice lacking sLe^x^ glycans. Previous research using the DKO mice showed that leukocytes from these mutant mice express no E- and P-selectin ligand activity and completely lack selectin-dependent cell adhesion [18]. We therefore hypothesized that the lack of glycan-dependent leukocyte recruitment in the DKO mice should result in the attenuation of leukocyte infiltration in asthma. As expected, eosinophils from the DKO mice almost completely disappeared from the BALF after repeated OVA administration, indicating the importance of sLe^x^ in the infiltration of eosinophils in the asthma model. These results are consistent with the prior research using PSGL-1-deficient mice [24] in which eosinophil recruitment was reduced in the asthma model, suggesting that the inhibition of glycan-selectin interaction would be a reasonable approach for the treatment of asthma [25].

Inhibition of glycan-selectin interaction can be achieved by either blocking the selectins or blocking the ligands for selectins. Efforts have been made to develop selectin inhibitors that block selectin-glycan dependent leukocyte rolling. An oligosaccharide analog of sLe^x^, called CY-1503, showed protective effects in a pig model of acute lung injury by interrupting the binding of sLe^x^ to P- and E-selectin [26]. However, it was proved to have poor efficacy in clinical trials of the reperfusion injury, which might result from the poor metabolic stability in vivo [27]. Non-oligosaccharide synthetic inhibitor TBC1269, also known as bimosiamose, is a pan-selectin antagonist against all the three types of selectins [28]. It showed therapeutic effects in a sheep model of allergic asthma [29]. However, the results of clinical trials using TBC1269 had diversity via the approach of drug administration. It reduced the late asthmatic reactions by 50% in asthma patients via the administration by inhalation [30], but showed no effects via intravenous administration [31]. Although the clinical trials of TBC1269 provided some promise as potential therapeutics for asthma, the overall strategy against selectins has been still challenging.

Although glycosylation of sLe^x^ on PSGL-1 plays an important role for the glycan-selectin interaction, PSGL-1 does not always function as a main ligand for E-selectin [25], which makes targeting sLe^x^ glycans more reasonable for drug development. In addition, P-selectin is temporally expressed on endothelial cells upon stimulation, which makes it hard to define the right time window to block P-selectin itself. After expression, P-selectin is internalized within a short time period by endocytosis and newly synthesized P-selectin is stored in Weibel–Palade bodies in endothelial cells [32]. In contrast, the sLe^x^ glycans are always expressed on the surfaces of effector cells such as eosinophils. Therefore, it is likely that blocking sLe^x^ glycans, the ligand for selectins, can be a more effective way for the treatment of asthma. Surprisingly, no such ligand inhibitors have been tested in clinical trials. 

Protein-based selectin ligand inhibitors are expected to have better metabolic stability and thus better protective effects, although such trials are still under development [25,27]. We hypothesized that our mAb F2 can be one of such drug discovery candidates against asthma. It binds to sLe^x^ with high specificity and showed strong suppressive effects in blocking eosinophil infiltration in our asthma model. The number of total leukocytes and eosinophils in the BALF was strongly suppressed in the F2-administered group. In addition, the results of the histological analysis suggested a relationship between eosinophil infiltration and allergic responses, including overproduction of mucin in the airway and fibrosis of the respiratory tracts. Blocking eosinophil infiltration also suppressed the allergic immune responses in the F2-administered mice. The levels of IgE in the serum along with the Th2 cytokine, eosinophil-related chemokines, and granule protein were suppressed, indicating that the allergic immune response caused by the intranasal challenge was relieved by F2 administration. The reduced production of the Th2 cytokine IL-5 after F2 administration was also confirmed at the protein level using ELISA. These findings indicate that targeting sLe^x^ using the mAb F2 in a murine asthma model resulted in its therapeutic effects.

One might argue that preventing eosinophil infiltration into the sites of inflammation would lead to eosinophilia in the blood and affect the function of other tissues. However, we observed that the deficiency or blockage of sLe^x^ resulted in a decrease in the percentage of eosinophils in the peripheral blood in the asthma model. As eosinophils are differentiated and proliferated in the bone marrow [33], we further investigated the eosinophils in the bone marrow in the allergic asthma model. As expected, the differentiation of eosinophils showed a decrease in the bone marrow in the DKO mice, and the WT mice treated with the mAb F2 after induction of asthma. Notably, IL-5 is responsible for the proliferation of eosinophils in the bone marrow and their translocation from the bone marrow into the blood [34]. In addition, previous research has shown that circulating IL-5, but not local IL-5, is required for the development of an allergic asthma model [35]. The immune cells, which can release IL-5, mainly include Th2 cells and innate lymphoid cells (ILC) [36]. It has been shown that mature eosinophils can promote Th2 cytokine production from Th2 cells in allergic situations [37]. Therefore, the blockage of eosinophil infiltration into the lungs in our study could have suppressed the release of IL-5 from Th2 cells. These results suggest that the deficiency or blockage of sLe^x^ locally can have a suppressive effect on eosinophil development in the bone marrow in asthma.

**Figure 7:**
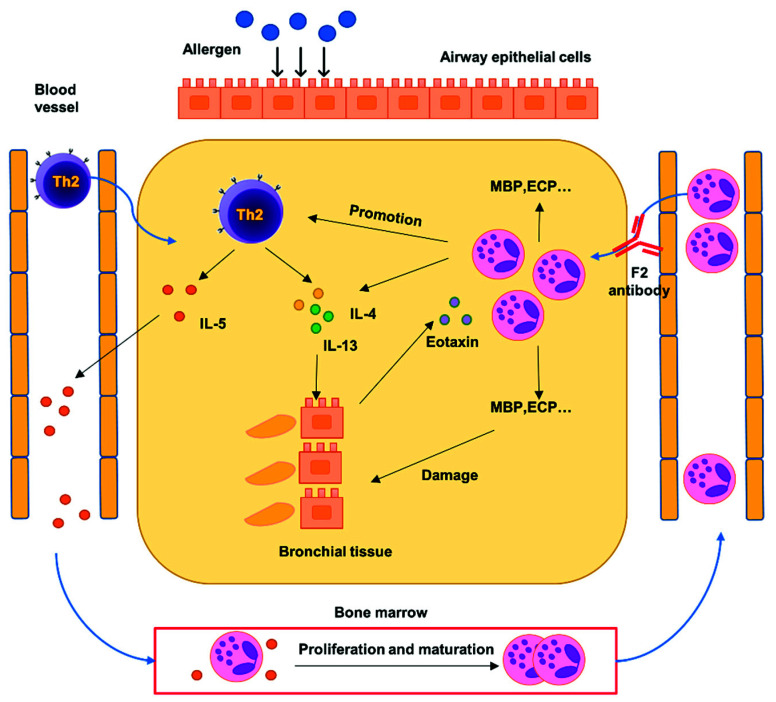
Possible mechanism underlying mAb F2-mediated suppression of the positive feedback loop in the asthma model. A hypothetical mechanism of the suppressive effects of F2 in the asthma model is shown in the figure. The initial exposure of the airway to environmental allergens can lead to the activation of pathogenic Th2 cells (upper left in purple). Th2 cells can then release Th2 cytokines, including IL-4, IL-5, and IL-13. IL-4 and IL-13 facilitate IgE class switching to activate local immune cells and cause damage to the bronchus. IL-5 is released into the peripheral blood and helps eosinophils to proliferate and mature in the bone marrow (bottom). The mature eosinophils translocate into the blood and infiltrate the sites of inflammation through the binding of sLe^x^ to P-selectin. The infiltrated eosinophils release tissue-damaging proteins such as MBP and cytokines, including IL-4 and IL-13. As a result, a positive feedback loop is formed, such that an increasing number of eosinophils are recruited to the lungs. However, when mAb F2 is administered (upper right), eosinophil infiltration is prevented in the lungs, which breaks the whole positive feedback loop to aggravate asthmatic symptoms, leading to a possible treatment for asthma.

Based on the results of this study, we hypothesized that a positive feedback loop is involved in the progression of allergic asthma (Figure 7). The initial part of the positive feedback loop might start from the inhalation of allergens, resulting in the activation of pathogenic Th2 cells in the lungs [38]. The activated Th2 cells would then release Th2 cytokines including IL-5, IL-4, and IL-13. IL-5 is released into the peripheral blood to induce the maturation and proliferation of the eosinophil precursors in the bone marrow [34]. IL-4 and IL-13 facilitate the class switching of B cells to release IgE [39], which binds to mast cells. After repeated antigen exposure, histamine is released by the mast cells to damage the epithelial cells and activate the alveolar macrophages to release the CCL11 [40], which attracts the eosinophils to the tissue. Eosinophils would then be recruited to the asthmatic lung through interaction with its sLe^x^ glycans and endothelial P-selectin to damage the airway epithelial cells by releasing the granule proteins such as MBP [41] and to promote the Th2 cells to release more IL-4, IL-5, and IL-13, which results in the amplification of the whole inflammatory cascade [42]. This form of positive feedback loop would result in the recruitment of more eosinophils to the lungs and worsen the inflammation. Our rolling assay proved that sLe^x^ is critical for the rolling of eosinophils on P-selectin expressing cells, so that the subsequent infiltration cascade [43] can be stopped by blocking the function of sLe^x^. Therefore, the results of this study collectively support the idea that the mAb F2 not only inhibits selectin-mediated infiltration of eosinophils into the asthmatic lung, but also breaks the cycle of the positive feedback loop in our OVA-induced asthma model.

Limitations of this research have to be mentioned. As previously reported, F2 also binds to a sulfated structure of sLe^x^, called 6-sulfo sLe^x^, expressed on the high endothelial venules (HEVs) in lymph nodes [17]. 6-sulfo sLe^x^ serves as a ligand for L-selectin, which is responsible for the lymphocytes homing to lymph nodes [44]. However, the effects of blocking L-selectin-ligand interactions could not be assessed in our present study, because F2 serves as a pan-selectin inhibitor which binds to a common glycan structure found in the ligands for L-, P- and E-selectins. Prior research based on L-selectin-deficient mice showed partially decreased airway hyperresponsiveness in a murine model of asthma, but the airway inflammation and leukocyte recruitment were unchanged in the KO mice compared with those in WT mice [45], indicating that L-selectin was not as important as P- and E-selectin in a murine model of allergic asthma. Although in a sheep model of asthma, a monoclonal antibody called MECA-79 reactive against sulfated sLe^x^ showed suppressive effects in both the airway hyperresponsiveness and L-selectin-mediated leukocyte recruitment through ectopically induced HEV-like blood vessels [46], this difference of L-selectin-dependency in the asthma models might be possibly due to the species difference. Considering the above as well as the results of our present study, we think it likely that the suppressive effects of F2 should be mainly mediated by blocking P- and E-selectin ligands, although the involvement of 6-sulfo-sLe^x^, a major ligand for L-selectin, in the asthma model needs further investigation.

Administration of the mAb F2 achieved great therapeutic effects in the mouse model of asthma. Since F2 is a mouse mAb, it cannot be directly applied to asthma patients unless it is humanized. However, this study strongly supports the idea that drug development based on the control of sLe^x^-dependent eosinophil infiltration could be applied as a novel treatment for allergic asthma.

## 4. Materials and Methods

### 4.1. Animals

Female C57BL/6J mice, aged 8–10 weeks, were purchased from Charles River Laboratories Japan and housed in the animal room of Chiba University. Mice doubly deficient in fucosyltransferase-IV (FucT-IV) and FucT-VII were generated and housed as previously described [18]. All mice were treated in accordance with the guidelines of the Chiba University Animal Care and Use Committee.

### 4.2. OVA-Induced Mouse Model of Asthma

For sensitization, the mice received intraperitoneal injection of OVA (100 μg per mouse dissolved in 200 μL sterile PBS, Grade V, Sigma-Aldrich, St. Louis, MO, USA) emulsified in 100 μL ImjectAlum (Thermo Fisher Scientific, Waltham, MA, USA) on days 0 and 7. The mice were anesthetized using 3% isoflurane and then challenged by intranasal administration of 100 μg OVA in sterile PBS, for 5 consecutive days starting on day 14. In the antibody administration experiments, 200 μg mAb F2, which was purified from ascites and examined as previously described [17], was administered intraperitoneally to each mouse on days 14, 16, and 18 before the immunization. The control mice received an intraperitoneal injection of 200 μL sterile-PBS.

### 4.3. Harvesting of Bronchoalveolar Lavage Fluid (BALF) and Lung Tissues

Twenty-four hours after the final intranasal challenge, the mice were anesthetized with sodium pentobarbital. The trachea was exposed and a 22G indwelling needle was inserted into it. The airway and lungs were washed with 1.5 mL ice-cold PBS supplemented with 2% FBS. The lung was filled with a 0.5 mL O.C.T. compound (Sakura, Tissue-Tek, Torrance, CA, USA) and stored at −80 °C until use. In some experiments, the lung was filled with 0.5 mL 10% formalin neutral buffer solution and then excised and fixed in 10 mL formalin for histological analysis. In other experiments, the lung was submerged in TRIzol^TM^ Reagent (Thermo Fisher Scientific, Waltham, MA, USA) and stored at −80 °C for total RNA extraction. 

### 4.4. Flow Cytometric Analysis of BALF, Blood and Bone Marrow

The BALF was filtered through a 70 μm nylon mesh and centrifuged at 440× *g* for 5 min at 4 °C. The supernatant was collected and stored at −80 °C for protein measurement. For the collection of bone marrow cells, the hind femur was dissected, and the cells were washed with 2 mL RPMI-1640 (Thermo Fisher Scientific, Waltham, MA, USA) using a 26 G needle. The red blood cells in the BALF and bone marrow were lysed using ammonium-chloride-potassium buffer (150 mM NH_4_Cl, 10 mM KHCO_3_, and 1 mM EDTA-2Na, pH 7.2). The remaining cells (leukocytes) were preincubated with anti-mouse CD16/32 antibody (Tonbo, San Diego, CA, USA, clone: 2.4G2) for 10 min at 4 °C to block the Fc receptors, and then with the following antibodies for 20 min at 4 °C: PE-Cy7-conjugated anti-mouse CD45 (BioLegend, San Diego, CA, USA, clone: 30-F11), APC-conjugated anti-mouse CD11c (BioLegend, San Diego, CA, USA, clone: N418), PE-conjugated anti-mouse Siglec-F (BD Biosciences, San Jose, CA, USA, Clone: E50-2440), FITC-conjugated anti-mouse CD4 (eBioscience, San Diego, CA, USA, Clone: RM4.5), PE-Cy7-conjugated anti-mouse CCR3 (BioLegend, San Diego, CA, USA, Clone: J073E5), and biotin-conjugated mAb F2. The samples were washed with PBS containing 0.1% BSA and centrifuged at 440× *g* for 5 min at 4 °C. The cells were incubated with BV421-conjugated streptavidin (BioLegend, San Diego, CA, USA, #405225) for 20 min at 4 °C, centrifuged at 440× *g* for 5 min at 4 °C, resuspended in 300 μL filtered-PBS and subjected to FACS analysis (Beckman Coulter, CytoFlex, Brea, CA, USA). The data were processed and analyzed using the FlowJo software version 10.6.1. (BD Biosciences, San Jose, CA, USA).

### 4.5. Enzyme-Linked Immunosorbent Assay (ELISA) for the Measurement of Serum IgE and IL-5

To detect OVA-specific IgE in mouse serum, the wells of a 96-well ELISA plate (Costar Assay Plate, half area, CORNING, Corning, NY, USA) were coated overnight with 2.0 μg/mL anti-mouse IgE (BioLegend, San Diego, CA, USA, clone: RME-1). After coating, the wells were blocked with 3% BSA in PBS for 1 h at room temperature. Diluted serum samples were added to the wells and incubated for 2 h. After washing with PBS containing 0.05% Tween 20, the wells were incubated with HRP-conjugated anti-mouse IgE (1:2000 dilution, Southern Biotech, Birmingham, AL, USA) for 1 h. After washing, 25 μL 1-step Ultra ELISA substrate (Thermo Fisher Scientific, Waltham, MA, USA) was added to the wells, and the optical density was measured at 450 nm using a 96-well spectrometer (Spectra Rainbow Thermo, TECAN, Menendorf, Switzerland). To determine the concentration of Th2 cytokine IL-4 and IL-5, the ELISA MAX Deluxe Set Mouse IL-4 (BioLegend, San Diego, CA, USA) and ELISA MAX Deluxe Set Mouse IL-5 (BioLegend, San Diego, CA, USA) were used, and all the steps were performed according to the manufacturer’s instructions. 

### 4.6. Real-Time Quantitative PCR

Total RNA was extracted from the upper lobe of the lung tissues using TRIzol reagent (Thermo Fisher Scientific, Waltham, MA, USA). cDNA was synthesized using ReverTra Ace qPCR RT Master Mix (Toyobo, Osaka, Japan), and the concentration was determined using NanoDrop One (Thermo Fisher Scientific, Waltham, MA, USA). Reverse Transcription quantitative PCR (RT-qPCR) was performed using the THUNDERBIRD SYBR qPCR Mix (Toyobo, Osaka, Japan). The expression of each mRNA was normalized to the expression of β-actin using the ΔΔCt method according to the manufacturer’s instructions (Thermal Cycler Dice TP870, Takara Bio Inc, Shiga, Japan). The primer sets used were as follows: β-actin, 5′-CATCCGTAAAGACCTCTATGCCAAC-3′ and 5′-ATGGAGCCACCGATCCACA-3′; IL-4, 5′-TCTCGAATGTAC-CAGGAGCCATATC-3′ and 5′-AGCACCTTGGAAGCCCTACAGA-3′; CCL11, 5′-TCCACAGCGCTTCTATTCCTG-3′ and 5′-TAAAGCAGCAGGAAGTTGGGA-3′; IL-5, 5′-AGCACAGTGGTGAAAGAGACCTT-3′ and 5′-TCCAATGCATAGCTGGTGATTT-3′; IL-13, 5′-AGACCAGACTCCCCTGTGCA-3′ and 5′-TGGGTC-CTGTAGATGGCATTG-3′; MBP, 5′-TTTGCAAACTTGACAAGACCCA-3′ and 5′-TGCATCCCTAGGCAAGTTCTC-3′.

### 4.7. Immunofluorescence

The lung tissue blocks frozen with the O.C.T. compound (Sakura, Tissue-Tek, Torrance, CA, USA) were cut into 12-µm-thick sections using a cryostat (HM525, Thermo Fisher Scientific, Waltham, MA, USA). For immunohistochemistry, the sections were fixed with ice-cold acetone, and the non-specific binding sites were blocked with PBS containing 3% BSA (Sigma-Aldrich, St. Louis, MO, USA) for 1 h. After washing, the sections were incubated overnight with the primary antibodies. The primary antibodies used were as follows: Alexa Fluor 488-conjugated anti-mouse CD45 (BioLegend, San Diego, CA, USA, Clone: 30-F11), Alexa Fluor 647-conjugated anti-mouse CD31 (BioLegend, San Diego, CA, USA, Clone:MEC13.3), Alexa Fluor 488-conjugated anti-mouse CD11c (BioLegend, San Diego, CA, USA, Clone: N418), and BV421-conjugated anti-mouse Siglec-F mAb (BD Biosciences, San Jose, CA, USA, clone: E50-2440). Sections stained with Alexa Fluor 488-conjugated anti-mouse CD45 and Alexa Fluor 647-conjugated anti-mouse CD31 mAbs were further incubated with 4′,6-diamidino-2-phenylindole dihydrochloride (DAPI) for 1 h. After incubation, the sections were mounted with Fluoromount (Diagnostic BioSystems, Pleasanton, CA, USA) and air-dried for over 30 min. For hematoxylin and eosin (H&E) staining, the sections were fixed with 10% buffered formalin for 5 min. After washing with PBS, the sections were stained with hematoxylin for 5 min and then with eosin for 5 min. The sections were dehydrated with gradient ethanol and xylene before mounting with Mount-Quick (Daido Sangyo, Tokyo, Japan). All images were obtained using a fluorescence microscope (BZ-9000; Keyence, Osaka, Japan). 

### 4.8. Histological Analysis

The lungs of mice were filled with 10% formalin (0.5 mL) after sacrifice. The collected lungs were fixed in 10 mL 10% formalin at room temperature overnight. The fixed lungs were washed twice with PBS and dehydrated for 1 h in the following order: 70%, 80%, 90% and 100% ethanol, and 100% xylene. After dehydration, the lungs were embedded in paraffin. Sections (2 μm) were cut and dried in air for 16 h at room temperature, and then deparaffinized in the order of 100% xylene, 100%, 90%, 80% and 70% ethanol. For H&E staining, the sections were stained with Mayer’s hematoxylin for 7 min and then with eosin for 5 min after washing with deionized water for 10 min. For Masson’s trichrome staining, the sections were stained with Weigert’s iron hematoxylin for 5 min, followed by washing in deionized water for 10 min. The sections were then stained with 0.8% Orange G solution for 10 min, Masson solution (Muto Pure Chemicals Co., Tokyo, Japan) for 7 min, 5% phosphotungstic acid solution for 15 min, and 2% Lightgreen solution (Muto Pure Chemicals Co., Tokyo, Japan) for 5 min. Between each step, the sections were washed with 1% acetic acid solution for 5 s. For periodic acid-Schiff staining, the sections were stained with 0.5% periodic acid solution for 10 min, followed by washing in deionized water. The sections were then stained with Schiff solution (Muto Pure Chemicals Co., Tokyo, Japan) for 20 min and sulfurous acid solution (Wako Co., Osaka, Japan) for 3 min three times. The sections were then stained with Mayer’s hematoxylin for 3 min, followed by washing in running water for 10 min. After staining, the sections were dehydrated in the following order: 70%, 80%, 90% and 100% ethanol, and 100% xylene before mounting with Mount-Quick (Daido Sangyo, Tokyo, Japan). All images were obtained using a fluorescence microscope (BZ-9000; Keyence, Osaka, Japan). A histology score was determined by the severity of leukocyte infiltration as follows: 0, normal; 1, less than 10 infiltrated leukocytes around the airway in the field; 2, 10 to 100 infiltrated leukocytes around the airway in the field; 3, moderate infiltration with a thin ring-shaped leukocyte infiltration (containing 100 to 1000 cells) around the airway in the field; and 4, severe infiltration with a thick ring-shaped leukocyte infiltration (containing more than 1000 cells) around the airway in the field captured using the 10× objective lens. Four fields were counted for each section and the histology score was calculated.

### 4.9. Ex Vivo Induction of Eosinophils

Bone marrow from C57BL/6J wild-type (WT) mice or FucT-IV/VII-doubly deficient (DKO) mice were collected as described above. Mouse hematopoietic progenitor cells were isolated from the bone marrow using the Mojosort^TM^ hematopoietic progenitor cell isolation kit (BioLegend, San Diego, CA, USA) according to the manufacturer’s instructions. The isolated cells were diluted to 10^5^ cells/mL with IMDM medium (Thermo Fisher Scientific, Waltham, MA, USA supplemented with 10% FBS (BioWest, Riverside, MO, USA), 10 mM HEPES (Sigma-Aldrich, St. Louis, MO, USA), and 100 U/mL Penicillin-100 μg/mL Streptomycin (Wako Co., Osaka, Japan) and incubated in 24-well plates. Next, 100 ng/mL mouse FLT3 ligand (BioLegend, San Diego, CA, USA, #579702) and 100 ng/mL mouse SCF (BioLegend, San Diego, CA, USA, #550704) were added to the culture medium on days 0 and 2. The medium was partly replaced with medium supplemented with 10 ng/mL mouse IL-5 (BioLegend, San Diego, CA, USA, #581502) on days 4, 7, and 10. Eosinophil induction was determined using flow cytometry. 

### 4.10. Eosinophil Rolling Assay

Mouse P-selectin (CD62P) cDNA was prepared by RT-PCR using total RNA from the mouse bone marrow and cloned into pcDNA3.1(+) Neo (Invitrogen, Waltham, MA, USA) expression vector. CHO-K1 cells were transfected with the expression vector, mouse P-selectin/pcDNA3.1(+) Neo, using Neon^TM^ Transfection System (Thermo fisher Scientific, Waltham, MA, USA), and cultured in the presence of 1.2 mg/mL G418 (Nacalai tesque, INC., Kyoto, Japan). After cloning by limiting dilution, cells expressing mouse P-selectin were selected by flow cytometry using anti-mouse CD62P antibody (BD Pharmingen^TM^, Franklin Lake, NJ, USA) and APC-conjugated anti-rat IgG F(ab’)_2_ (Santa Cruz Biotechnology, Dallas, TX, USA). CHO-K1 cells stably expressing mouse P-selectin were then cultured as monolayers in 35-mm culture dishes (Corning, Corning, NY, USA). Bone marrow-derived eosinophils were fluorescently labeled with 1 μM CFSE (Thermo Fisher Scientific, Waltham, MA, USA, Molecular Probes^®^, Eugene, OR, USA) at 37 °C for 20 min and incubated with or without 10 μg/mL purified F2 for 10 min at room temperature. After incubation, the dishes were equipped with a parallel plate flow chamber (GlycoTech Co., Gaithersburg, USA), according to the manufacturer’s instructions. CFSE-labeled eosinophils from the WT and DKO mice were resuspended in the rolling assay buffer (20 mM HEPES-NaOH, 150 mM NaCl, 1 mM MgCl_2_, 1 mM CaCl_2_, and pH 7.4) containing 0.1% BSA at a density of 4 × 10^6^ cells/mL and introduced into the flow chamber at a wall shear stress of 3.0 dynes/cm^2^ using a syringe pump Model 11 Plus (Harvard Apparatus Co., Holliston, MA, USA). Images were captured using an OptiMOS^TM^ Scientific CMOS camera (QImaging Co., Kent, UK) equipped with an inverted microscope (Zeiss Axiovert S100, White Plains, NY, USA) and analyzed using Fiji ImageJ. 

### 4.11. Migration Assay

Bone marrow cells were prepared as described above and diluted to 10^6^ cells/mL. The diluted cells were incubated with PBS or 10 μg/mL F2 in PBS on ice for 20 min. Five hundred μl RPMI-1640 (Thermo Fisher Scientific, Waltham, MA, USA) was added to each well of the 24-well plate. Recombinant murine CCL11 (Biolegend, San Diego, CA, USA) was added to the medium at the concentration of 50 ng/mL. The uncoated transwell was inserted into each well carefully. After applying the cell suspension (2 × 10^5^ cells per well), the 24-well plate was incubated at 37 °C for 4 h. After incubation, the number of eosinophils in the lower chamber was analyzed by flow cytometry.

### 4.12. Statistical Analysis

The data are presented as the mean ± SEM, as indicated in the figure legends. The statistical significance of differences was assessed using the Student’s t-test. Comparison including three groups was analyzed with one-way analysis of variance (ANOVA) and post hoc Tukey’s HSD test. Statistical significance was set at *p* < 0.05. Statistical analyses were performed using GraphPad Prism 7.0.

## Data Availability

The data presented in this study are available on request from the corresponding author.

## Supplementary Materials

The following are available online at https://www.mdpi.com/article/10.3390/ijms22189961/s1, Vidio S1: Rolling of untreated eosinophils from the WT mice, Vidio S2: Rolling of mAb F2-treated eosinophils from the WT mice, Vidio S3: Rolling of eosinophils DKO mice.
